# Clinico-Hematological and cytogenetic spectrum of adult myelodysplastic syndrome: The first retrospective cross-sectional study in Iranian patients

**DOI:** 10.1186/s13039-021-00548-z

**Published:** 2021-05-08

**Authors:** Mostafa Paridar, Kazem Zibara, Seyed Esmaeil Ahmadi, Abbas Khosravi, Maral Soleymani, Ebrahim Azizi, Omid Kiani Ghalesardi

**Affiliations:** 1 Deputy of Education, Ministry of Health and Medical Education, Tehran, Iran; 2 Department of Hematology and Blood Banking, School of Allied Medicine, Iran University of Medical Sciences, Tehran, Iran; 3 PRASE, Biology Department, Faculty of Sciences-I, Lebanese University, Beirut, Lebanon; 4 Transfusion Research Center, High Institute for Research and Education in Transfusion Medicine, Tehran, Iran; 5 Research Center for Thalassemia and Hemoglobinopathy, Health Institute, Ahvaz Jundishapur University of Medical Sciences, Ahvaz, Iran; 6 Student Research Committee, Ahvaz Jundishapur University of Medical Sciences, Ahvaz, Iran

**Keywords:** Myelodysplastic syndromes, Hematological spectrum, Cytogenetic, Iran

## Abstract

**Background:**

Myelodysplastic syndrome (MDS), a heterogeneous group of hematopoietic malignancy, has been shown to present different cytogenetic abnormalities, risk factors, and clinico-hematological features in different populations and geographic areas. Herein, we determined the cytogenetic spectrum and clinico-hematological features of Iranian MDS patients for the first time.

**Methods:**

This prospective cross-sectional study was conducted on 103 patients with MDS in Ahvaz, southwest of Iran, from 2014 to 2018. Clinical presentations, complete blood counts (CBC), and bone marrow (BM) biopsy samples were assessed. Perls' staining was used to evaluate BM iron storage. The cytogenetic evaluation was performed using the conventional G banding method on the BM.

**Results:**

Patients’ median age was 62.3 (ranged from 50–76), and the majority were male (72.8%). The most common clinical symptom at the time of admission was fatigue (n = 33) followed by pallor (n = 27). The most common subgroup was MDS-Multi Lineage Dysplasia (MDS-MLD) (n = 38, 36.8%), followed by MDS-Single Lineage Dysplasia (MDS-SLD) (n = 28, 18.4%). A normal karyotype was observed in 59 patients (57.3%), while 44 patients (42.7%) had cytogenetic abnormalities. Trisomy 8 (+ 8) was the most common cytogenetic abnormality (n = 14) followed by del 17p (n = 9) and monosomy 7 (− 7) (n = 7). Twelve patients (11.65%) were transformed to AML.

**Conclusion:**

Our data betokened that among our MDS patients, Trisomy 8 is the predominant cytogenetic abnormality, and MDS-MLD and MDS-SLD are the most common of subtypes. Noteworthy, the male: female ratio was slightly higher in Iran than in previous reports from other parts of the world. Our study is the first report of the clinical, hematological, and cytogenetic spectrum of MDS patients in Iran

## Introduction

Myelodysplastic Syndromes (MDSs) are a group of hematopoietic disorders characterized by ineffective hematopoiesis, dysplasia of hematopoietic cells, and cytopenia in one or more blood cell lineages [1]. MDS generally occurs de novo; however, a small number of MDSs are secondary to radiation and/or chemotherapy exposure. Pathophysiology of the disease includes multi-step processes involving chromosomal abnormalities and/or genetic aberrations [2]. In the United States, MDS incidence is 3–4 per 100,000 annually and increases with age [3]. Usually, this disease is more prevalent in men than women, except for the 5q deletion subgroup, which is more common in women [4]. The disease course is very variable, ranging from an indolent to an aggressive state. However, it is often associated with an increased risk of acute myeloid leukemia (AML) transformation and a short survival rate [5]. Diagnosis of MDS transpires via scrutiny of the peripheral blood smear (PBS) and the bone marrow (BM). Pancytopenia and single or multilineage dysplasia are observed in the PB, whereas less than 20% blasts and hematopoietic cells dysplasia are detected in the BM. Based on the 2016 classification of the World Health Organization (WHO), MDS comprises six subgroups, including MDS with multilineage dysplasia (MDS-MLD), MDS with single lineage dysplasia (MDS-SLD), MDS with ring sideroblasts (MDS-RS), MDS with excess blasts (MDS-EB − 1 and 2), MDS with isolated del (5q), and unclassifiable MDS (MDS-U) [6].

Cytogenetic abnormalities such as amplifications, deletions, and translocations have been detected in about 50% of patients with de novo MDS and 80% of patients with secondary MDS, as well [7]. Evaluation of cytogenetic abnormalities is useful in the diagnosis of MDS and crucial in determining the prognosis [8]. Indeed, the chromosomal abnormality type is one of the most important factors employed in the MDS Revised International Prognostic Scoring System (R-IPSS) [9]. Patient prognosis helps determine the disease's clinical course and plays a vital role in establishing the patient's therapeutic plan [10].

There are many reports from western countries in terms of cytogenetic profiles of MDS patients. However, clinical symptoms, cytogenetic abnormalities, and the disease's pathophysiology are dissimilar in different populations and vary greatly in different geographic areas. Therefore, the current study aimed to determine the cytogenetic spectrum and clinico-hematological features of MDS patients in the Khuzestan province, southwest of Iran. This is the first comprehensive report from Iranian MDS patients.

## Material and methods

### Patients and study design

This prospective cross-sectional study was conducted on 103 patients with MDS referred to Shafa Hospital in Ahvaz, southwest of Iran, from 2014 to 2018. Patients diagnosed as de novo MDS according to their clinical and hematological findings were included in the study. Notably, we excluded patients with cytopenia due to other non-malignant disorders and patients with a history of chemotherapy or radiotherapy. All patients were diagnosed and classified according to the classification of myeloid neoplasms and acute leukemia of the 2016 revision of the World Health Organization [6]. This study was approved by the Ethics Committee of Ahvaz Jundishapur University of Medical Sciences. Signed consent forms were received from all the patients prior to participation in the study.

### Hematological assessments

Following the clinical examination, complete blood counts (CBC) were performed, and PBS was scrutinized. Patients suspected of having MDS were subjected to further evaluation by BM biopsy samples obtained from the posterior superior iliac spine. According to the manufacturer's protocol, assessment of Iron was performed on the BM using the Perls' stain kit (Sigma-Aldrich).

### Cytogenetic analysis

The cytogenetic evaluation was performed using a conventional G banding method on the BM samples. First, BM cells were cultured for 24 h in 5 ml of Marrowmax medium, 20% FBS, 2 mg L-glutamine, and 100 ml/U penicillin/streptomycin. Afterward, a total of 50 μl of colcemid (10 μg/ml) was added to the culture medium, and cells were incubated for 20 min at 37 ºC. Following this, a hypotonic potassium chloride solution was added to the cells which were incubated for 25 min at 37 ºC. CELLS were then fixed with methanol:acetic acid (3:1), and slides of metaphase chromosomes were prepared in a humidified and controlled temperature environment. Metaphase chromosomes (at least 20 metaphases) were banded using the Giemsa trypsin banding (GTG) method, and karyotypes were defined according to the International System for Human Cytogenetic Nomenclature (ISCN) 2013 criteria.

### Statistical analysis

Data are displayed as mean values, while the standard error of the mean was shown using error bars (Mean ± SEM). GraphPad Prism (Version 8.0.1.244, San Diego, CA, USA) software was utilized to perform statistical analyses.

## Results

### Patients characteristics

Among 300 patients originally referred to the hospital, a total of 103 patients were diagnosed as de novo MDS according to their clinical and hematological tests and hence were included in the study. Patients’ median age was 62.3 years (50–76), and the majority of patients were of male (72.8%). The mean levels of hemoglobin (Hb), platelets (plt), and white blood cells (WBC) were 9.9 g/dl, 103 × 10^3^/μl, and 3.4 × 10^3^/μl; respectively (Table 1).

**Table 1:** Patients’ characteristics

Gender	Male	75 (72.81%)
	Female	28 (27.19%)
Age (years)	Median (range)	62.3 (50–76)
Work history	Agriculturist	23 (22.33%)
	Petroleum worker	7 (6.79%)
	Truck driver	5 (4.85%)
	Others	68 (66%)
Hemoglobin (g/dl)	Median ± SD	9.9 ± 1.1
Platelet (× 10^3^/μl)	Median ± SD	103 ± 20
WBC (× 10^3^/μl)	Median ± SD	3.4 ± 0.5
ANC (× 10^3^/μl)	Median ± SD	1.6 ± 0.3
BM cellularity	Hypercellular	78 (75.73%)
	Normocellular	18 (17.47%)
	Hypocellular	7 (6.8%)
BM Iron storage	Increased	86 (83.5%)
	Decreased	0
	Normal	17 (16.5%)

*WBC* white blood cells, *ANC* absolute neutrophil count, *BM* bone marrow

More than 75% of the patients presented hypercellular BM. Moreover, iron storage was increased in 86 cases (83.5%) (Table 1). The major clinical symptom at the time of admission was fatigue (n = 33) followed by pallor (n = 27), easy bruising and bleeding (n = 20), recurrent infections (n = 19), and bone pain (n = 4). Finally, a total of twelve patients (11.65%) were transformed to AML. Based on FAB classification, 4, 6, and 2 patients progressed to AML-M0, AML-M1, and AML-M2, respectively.

### Patients classification

The patients were classified into different subgroups according to the 2016 revision of the WHO classification. The most common subgroup was MDS-MLD (n = 38, 36.8%) followed by MDS-SLD (n = 28, 18.44%). However, the rarest subgroup was MDS-RS (n = 3, 2.91%) (Table 2).

**Table 2:** Patients’ subgroups based on WHO Classification 2016

Subgroups		Number of patients	Normal cytogenetics	Abnormal cytogenetics
MDS-MLD		38 (36.89%)	22	16
MDS-SLD		28 (18.44%)	17	11
MDS-EB	MDS- EB-1	15 (14.56%)	11	4
	MDS-EB -2	11 (10.67%)	5	6
MDS with isolated del(5q)		4 (3.88%)	0	4
MDS-RS		3 (2.91%)	1	2
MDS-U		4 (3.88%)	3	1

*MDS-MLD* MDS with multilineage dysplasia, *MDS-SLD* MDS with single lineage dysplasia, *MDS-EB* MDS with excess blasts, *MDS-RS* MDS with ring sideroblasts, *MDS-U* MDS unclassifiable

### Patients karyotypes

**Fig. 1:**
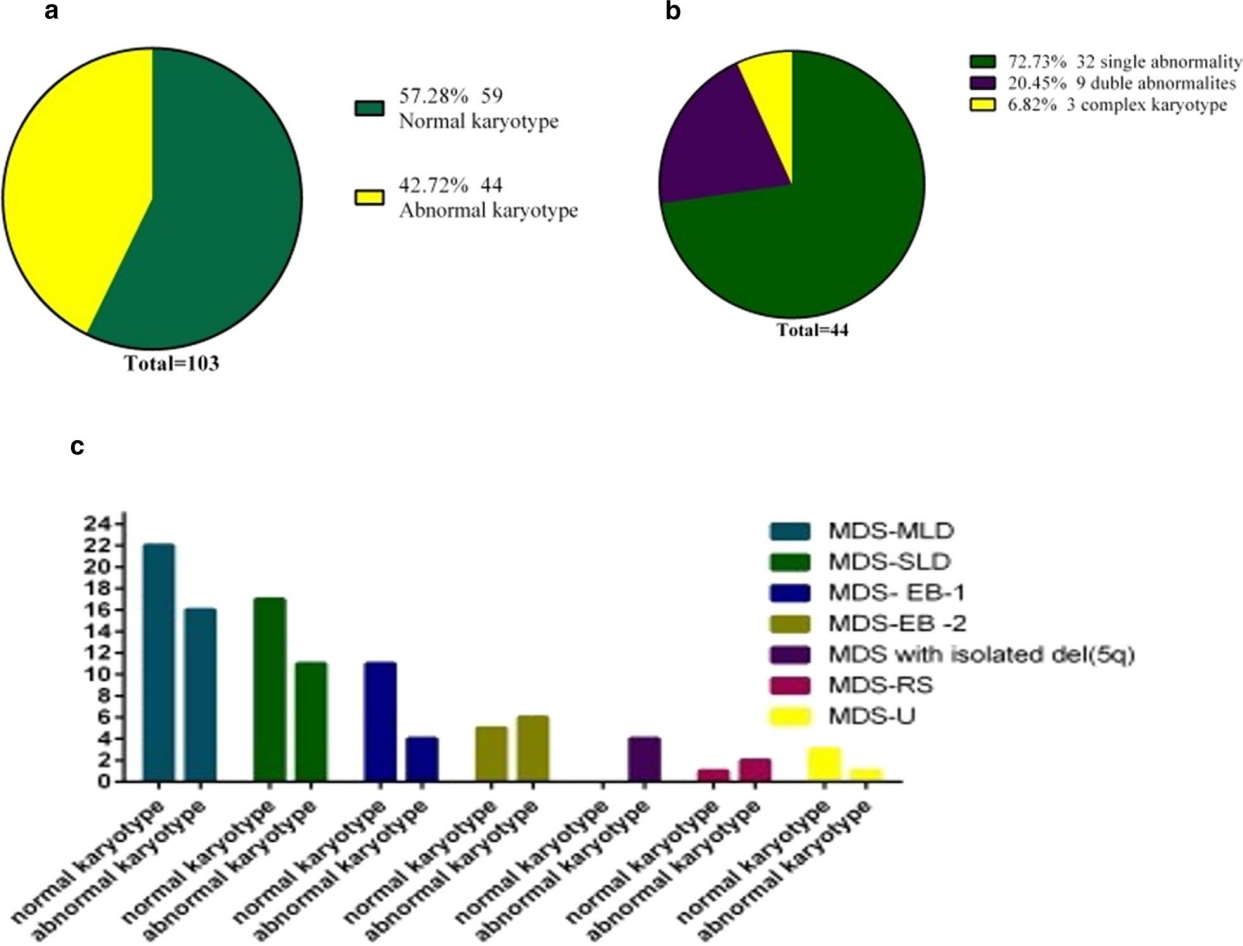
Prevalence of the cytogenetic abnormalities observed in MDS patients. **a** Prevalence of normal and abnormal karyotypes among MDS patients. **b** Types of abnormalities among patients with abnormal karyotype. **c** Distribution of normal and abnormal karyotypes among MDS subgroups

**Fig. 2:**
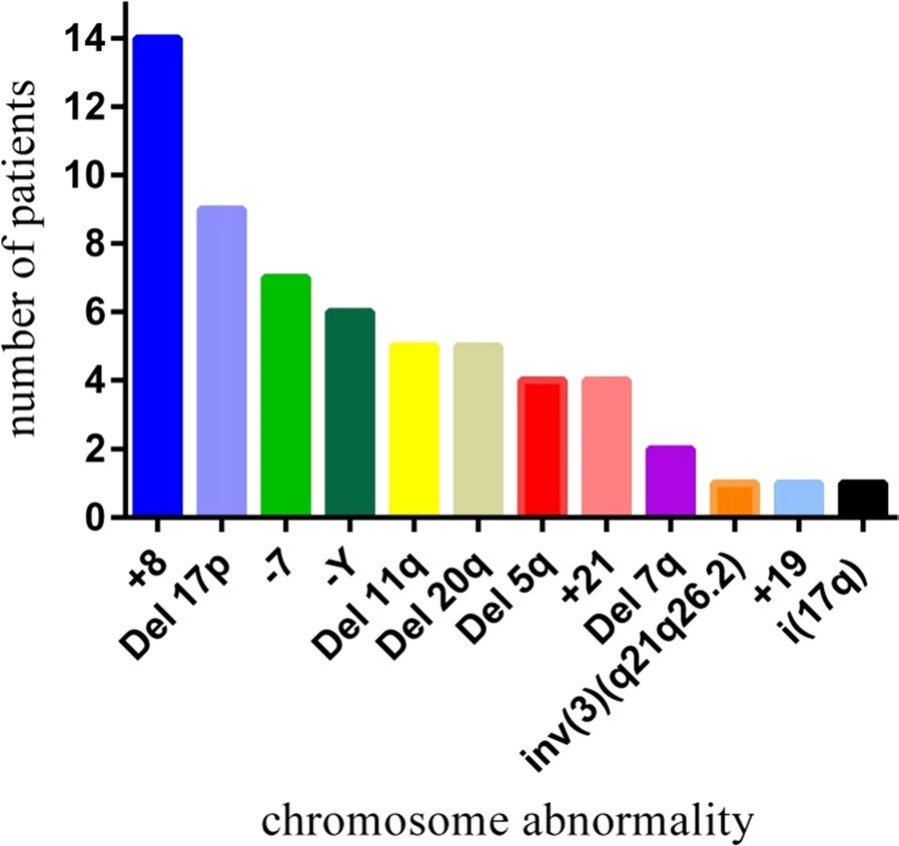
The prevalence of chromosomal abnormalities in MDS patients

Concerning the karyotypes of the patients, a total of 59 patients (57.28%) presented a normal karyotype, while the remaining 44 patients (42.72%) had cytogenetic abnormalities (Fig. 1a). Patients with abnormal karyotype showed different types of abnormalities. Single, double and complex abnormalities were observed in 32 (72.7%), 9 (20.5%) and 3 (6.5%) patients; respectively (Fig. 1b). The distribution of normal and abnormal karyotypes among MDS subgroups is demonstrated in Fig. 1c. On the other hand, the most common cytogenetic abnormality among the 44 MDS patients was Trisomy 8 (+ 8) (n = 14), followed by del 17p (n = 9) and monosomy 7 (− 7) (n = 7) (Fig. 2). The prevalence and distribution of the chromosomal abnormalities in MDS patients are detailed in Fig. 2.

### Patients prognosis

The patients’ prognosis was assessed according to the Revised International Prognostic Scoring System [R-IPSS]. Results showed that a total of 68 patients (66%) were considered as good or very good prognosis (Table 3). Moreover, 26 cases (25.2%) showed an intermediate prognosis. However, a poor prognosis was observed in 9 cases (8.7%), while none of the patients showed very poor prognosis (Table 3).

**Table 3:** Cytogenetic score groups based on R-IPSS

Cytogenetic prognosis subgroup	Cytogenetic abnormality	Total	MDS subtypes						
			MDS-SLD	MDS-MLD	MDS-EB-1	MDS-EB-2	MDS with del (5q)	MDS-RS	MDS-U
Very good	-y, del 11q	3	2 (7.1%)	1 (2.6%)	0	0	0	0	0
Good	Normal	59	17 (60.7%)	22 (57.9%)	11 (73.3%)	5 (43.5%)	0	1 (33.3%)	3 (75%)
	del5q, del20q	6	1 (3.5%)	1 (2.6%)	0	0	4 (100%)	0	0
Intermediate	+ 8, + 19, del 7q, i(17q), other single/double abnormalities	26	8 (28.6%)	11 (28.9%)	3 (20%)	1 (9.1%)	0	2 (66.7%)	1 (25%)
Poor	-7, inv (3), double including − 7/del(7q) and complex (3 abnormalities)	9	0	3 (7.9%)	1 (6.6%)	5 (45.5%)	0	0	0
Very poor	> 3 abnormalities	0	0	0	0	0	0	0	0

## Discussion

MDS is a heterogeneous clonal disorder associated with ineffective hematopoiesis, reduced blood cells, and hematopoietic cells' dysplasia. Generally, MDS is known as a preleukemic disorder and is considered an elderly disease. Indeed, the mean age of patients in our study was 66 years, with a range of 50–76 years, which is similar to MDS in the US population (67 years) [11], and close to Turkey (69 years) [12] and European countries such as Germany (70 years) [13] and Poland [14] (70 years). On the other hand, our results are significantly different from those of East Asian countries such as China (49 years) [15], India (42, 45, and 55 years) [16–18], and Japan (76 years) [19]. Most of the patients were farmers or worked in petroleum jobs. It is worth noting that most of the farmers in the southwest regions of Iran, where our study was conducted, are exposed to pesticides. The association of pesticide exposure and MDS incidence was recently confirmed in a meta-analysis study [20]. Moreover, many investigators reported that petroleum workers exposed to benzene had an important MDS risk factor [21, 22]. The male to female ratio was 2.6:1, which was clearly higher than those reported from the US (1.9: 1) [11], Korea (1.7: 1) [23], China (1.3: 1) [15], and Pakistan (1.7: 1) [24]. In Iranian culture, women do not work as much as men, especially the high-risk jobs, which might explain the higher male: female ratio in our study compared to other reports.

The mean hemoglobin level in our study on southwest Iranian patients was 9.9 g/dl, which is similar to that obtained in Turkey (9 g/dl) [12] and Greece (9.5 g/dl) [25]. However, our hemoglobin data are higher than those obtained in Pakistan (7.7 g/dl), China (6.3 g/dl) [15], Singapore (7.7 g/dl) [26], and India (6.84 g/dl) [18]. The difference may be due to the higher percentage of males in our study and the fact that Iran implements a national iron supplementation program. On the other hand, the mean platelet counts in our study was 103 × 10^3^/μl, which is similar to those obtained by Lau et al*.* in Singapore (101 × 10^3^/μl) [26]. However, our data on platelets counts are higher than those obtained by Chen et al*.* in China (42 × 10^3^/μl) [15], Ehsan et al*.* in Pakistan (60 × 10^3^/μl), and Chaubey et al. in India (85 × 10^3^/μl)[18]. Noteworthy that even higher platelet counts were reported in Turkey (163 × 10^3^ μl) [12] and Greece (158 × 10^3^/μl) [25].

Although our study included patients from the 2014 to 2018 period, all patients were re-classified according to the 2016 revision of the World Health Organization classification of myeloid neoplasms and acute leukemia [6]. Our data showed that the most common MDS subgroup was MDS-MLD (36.9%), followed by MDS-SLD (18.4%). These results are in line with those of Haase et al*.* in Germany [27] and Rashid et al*.* in Pakistan [28], who reported that MDS-MLD is the most common subtype (27.6% and 52.1%, respectively). Moreover, Pozdnyakova et al*.* in the USA [11] and Li et al*.* in China [29] reported that MDS-MLD was the most common subgroup (Respectively 32.2% and 44%) followed by MDS-EB-I. In contrast, Chauby et al*.* reported MDS-SLD as the most common type of MDS in India [18]. The aforementioned differences might be due to the different ethnicity, population of the study, and/or sensitivity of the applied laboratory assay.

Cytogenetic abnormalities play a substantial role in the pathogenesis of MDS and are key factors in the diagnosis, classification, and prognostic scoring of the disease. However, the pathogenesis of MDS is still not well understood in Iran. The environmental, occupational, and genetic factors in Iran's southwest region are very different from western countries. This would affect the pathogenesis of MDS and may cause various chromosomal abnormalities, different frequencies, and patterns of MDS subtypes. Chromosomal abnormalities were observed in 42.7% of the patients, in accordance to other studies conducted in different parts of the world such as in Pakistan (42.3%) [28], the United States (44.5%) [11], Germany (49.8%) [27], Tunisia (51%) [30], and India (47.5%) [18]. However, this was less than what was found in China (67.5%) [29]. In particular, 41.7%, 29.5%, and 28.8% of the reported chromosomal abnormalities were related to copy number only, structural abnormalities, and concomitant occurrence of both, respectively [29]. Among patients with abnormal karyotypes, 72.7% had a single abnormality, 20.5% had double abnormalities, while 6.8% had complex abnormalities (more than 3 abnormalities). The prognostic score of the studied patients was assessed according to R-IPSS. Accordingly, more than 91.3% of patients had very good, good, and intermediate prognosis. However, the poor prognosis was observed in only 9 cases, and very poor prognosis was observed in none of the patients.

In this study, the most common chromosomal abnormality was trisomy 8 (13.6% of all patients and 31.8% of patients with abnormal karyotype), followed by del 17p (8.7% of all patients and 20.5% of patients with abnormal karyotype). Trisomy 8 was observed in 10 patients as a single chromosomal abnormality, in 2 patients with double chromosomal abnormalities, and in 2 patients with a complex karyotype. In accordance, Li et al*.* in China [29] and Rashid et al*.* in Pakistan [28] showed that the most common chromosomal abnormality was trisomy 8. In contrast, the most common chromosomal abnormality in Tunisia [30], Germany [27], Switzerland [31], Greece [25], and the United States [11] was − 5/del (5q). Our results showed that chromosomal abnormalities in Iranian MDS patients often include changes in the copy number (except for 2 cases), including deletion of all or part of a chromosome (e.g., del 17p or monosomy 7) or gain of a chromosome (e.g., trisomy 8), while balance abnormalities (such as translocations) have a very low incidence in patients. The changes in copy numbers result in a change in the dosage of genes, which can lead to the inactivation of tumor suppressor genes and the activation of oncogenes by various mechanisms such as haploinsufficiency and loss of heterozygosity, thus playing an essential role in the initiation and progression of the disease. For instance, it has been shown that the CUX1 gene, expressed on chromosome 7, acts as a tumor suppressor gene in myeloid precursors, decreases in MDS (and AML) with − 7/del (7q), and is involved in the pathogenesis of the disease [32]. It is worth noting that MDS cells with trisomy 8 (+ 8) express high levels of antiapoptotic proteins and have high resistance to apoptotic agents, such as gamma rays [33].

There has not been such a registry developed to capture the MDS epidemiology in Iran yet. So, we will be the pioneer to report the Clinico-Hematological and cytogenetic spectrum of adult myelodysplastic syndrome in addition to the plethora of scientific information that is already available in the western and eastern literature. It is worth mentioning that cytogenetics mainly aims to identify recurrent karyotypic alterations which are termed clonal chromosomal aberrations (CCAs), while non-clonal chromosome aberrations (NCCAs) might also play an intriguing role [34, 35]. Recently NCCAs have drawn more attention, so it would be interesting to include NCCA data for future studies, especially for patients who did not display clonal chromosome aberrations.

## Conclusion

Our data indicated that MDS-MLD and MDS-SLD are the most common subtypes of MDS in Iran's southwest region. In addition, Trisomy 8 was the most predominant cytogenetic abnormality. Moreover, our results showcased that the male: female ratio is slightly higher than previous reports from other parts of the world. This study represents the first report of the clinical, hematological, and cytogenetic spectrum of MDS patients in Iran. This study's major limitations are that patients’ overall survival, treatment strategies, and response to treatment were not evaluated and suggested to be resolved in future studies.

## Data Availability

The datasets used and analyzed during this study are available from the corresponding author on reasonable request.

## Abbreviations

MDS: Myelodysplastic syndrome
CBC: Complete blood counts
BM: Bone marrow
AML: Acute myeloid leukemia
PBS: Peripheral blood smear
WHO: World Health Organization
MDS-MLD: MDS-multi lineage dysplasia
MDS-SLD: MDS-single lineage dysplasia
MDS-RS: MDS with ring sideroblasts
MDS-EB: MDS with excess blasts
MDS-U: Unclassifiable MDS
R-IPSS: Revised international prognostic scoring system
GTG: Giemsa trypsin banding
ISCN: International system for human cytogenetic nomenclature
